# Visual and bibliometric analysis of chronic rhinosinusitis and nasal polyps

**DOI:** 10.1016/j.jacig.2024.100211

**Published:** 2024-01-18

**Authors:** Zhichen Liu, Yuxu Yao, Huanxia Xie, Aina Zhou, Yuhui Fan, Jisheng Liu, Qingqing Jiao

**Affiliations:** a Department of Ear, Nose, and Throat, The First Affiliated Hospital of Soochow University, Suzhou, China; b Department of Dermatology, The Second Affiliated Hospital of Soochow University, Suzhou, China; c Department of Dermatology, The First Affiliated Hospital of Soochow University, Suzhou, China

**Keywords:** Bibliometrics, Bibliometrix, chronic rhinosinusitis, nasal polyps, VOSviewer

## Abstract

**Background:**

Chronic rhinosinusitis (CRS) is a heterogeneous disease characterized by persistent sinonasal inflammation and sinus microbiome dysbiosis. Nasal polyps (NPs) are one of the main manifestations that cause diverse clinical symptoms of CRS.

**Objective:**

We sought to conduct a bibliometric and visual analysis of articles on CRS and NPs published between 2003 and 2022 to provide researchers with the current state of research and potential directions.

**Methods:**

We searched relevant articles from 2003 to 2022 in the Web of Science database. VOSviewer and the Bibliometrix R package were used to perform the bibliometric analysis.

**Results:**

A total of 3907 publications were retrieved. The United States made the highest contributions to global research, followed by China. Northwestern University had the most publications. The most published author was C. Bachert, followed by R. P. Schleimer and R. J. Schlosser. The authors with the most co-citations were C. Bachert, W. J. Fokkens, and P. Gevaert. Moreover, the journal with the most publications was the *International Forum of Allergy & Rhinology*, and the *Journal of Allergy and Clinical Immunology* was the most cited. “Covid-19,” “biologics,” and “type 2 inflammation” were the top current research hotspots.

**Conclusions:**

The United States and Northwestern University were the leading country and institution in researching CRS and NPs. C. Bachert was the most influential expert. The *International Forum of Allergy & Rhinology* and the *Journal of Allergy and Clinical Immunology* were leading journals. “Covid-19,” “biologics,” and “type 2 inflammation” were the trending topics.

Chronic rhinosinusitis (CRS) is a heterogeneous disease characterized by persistent sinonasal inflammation and sinus microbiome dysbiosis, affecting 5% to 12% of the population of most countries.^1^ Nasal polyps (NPs) are one of the main manifestations that cause diverse clinical symptoms of CRS. Patients with aspirin-exacerbated respiratory disease in Europe and the United States are more likely to present with NPs.^1^ As one of the most common diseases in most countries, the disease seriously affects the quality of patients’ daily life and burdens society and families. The dominant symptoms of CRS are nasal congestion, mucopurulent nasal discharge, facial pain/pressure/fullness, and decreased sense of smell.^1^ Currently, the primary treatment modalities include nasal or systemic glucocorticoid, antibiotics, aspirin desensitization, and endoscopic sinus surgery.^1^, ^2^, ^3^, ^4^, ^5^ New treatment modalities, such as biologics, have emerged in recent years, and clinical research has progressed rapidly. Therefore, it is essential to grasp the current status and potential research directions.

Bibliometrics is a statistical method to identify and summarize articles on a selected area in a systematic manner. Many medical fields now use bibliometrics for in-depth analysis.^6^, ^7^, ^8^, ^9^, ^10^, ^11^ It is helpful for researchers in related fields to accurately grasp the current research trends by analyzing the publication. Recently, multiple bibliometric software tools have been developed, such as VOSviewer,^12^^,^^13^ CiteSpace,^14^^,^^15^ and Bibliometrix R package.^16^^,^^17^ Researchers can analyze, compare, and visualize results across these tools and obtain information, including authors, countries, institutions, journals, and references, in the area of interest. Some researchers discussed the treatment of CRS until 2020 using bibliometrics.^18^ However, we found that the hotspots in the field had changed substantially in 2022. Therefore, this article provides the most popular research directions for CRS and NPs in recent years. In this study, we used the Web of Science database to perform a bibliometric analysis of articles related to CRS and NPs from 2003 to 2022 and used VOSviewer and the Bibliometrix R package to perform a visualization analysis. We summarized the significant contributors of CRS and NPs in the past 20 years and discussed the trending topics and hotspots in this field. We hope this article will reference the researchers and drive progress in the research of CRS and NPs.

## Methods

### Search strategy

The Web of Science database (https://www.webofscience.com/wos/woscc/basic-search) was used as the data source. The retrieval time of databases was as of January 2023. The search formula was (TS=(“chronic rhinosinusitis”) OR TS=(“chronic sinusitis”) OR TS=(“chronic nasosinusitis”)) AND (TS=(“nasal polyp”) OR TS=(“nasal polyps”) OR TS=(“nasal polyposis”)), LA = (English), and the type of documents was set to “articles” and “review.”

### Data analysis

Considering the respective properties and advantages, we simultaneously used VOSviewer and the Bibliometrix R package.

VOSviewer (version 1.6.18) is a bibliometric analysis software that can be used for network construction and visualization on the basis of publications, countries, institutions, journals and co-cited journals, authors and co-cited authors, or keywords.^12^^,^^13^ In the visual network diagram built by this software, a circle represents an analytical item, such as a country, institution, journal, or author. The size of the circle depicts the number of this item. Circles’ colors represent different clusters. The thickness of the lines between the circles reflects the degree of cooperation or co-citation between the analyzed items. The Bibliometrix R package (version 4.2.1) (https://www.bibliometrix.org) was used to conduct the bibliometric and visualized analysis. Besides, we used Bibliometrix to build the Bradford law diagram, publications’ geographic distribution maps, trend topic analysis, and the Sankey diagram.^14^^,^^15^

Microsoft Office Excel 2019 was used to graph the quantitative analysis of the publication. In addition, some inclusion thresholds were set during data analysis to filter out the more valuable results.

Furthermore, we analyzed the core authors in this field according to the Price law（*m*_p_ = 0.749 × $\sqrt{n_{\text{pmax}}}$). In this law, *m*_p_ is the minimum number of articles published by core authors in the statistical period, and *n*_pmax_ is the number of articles published by the authors with the most significant number of publications in the statistical period.

### Data statement

Raw data were obtained from the Web of Science database. The original contributions presented in the study are included in Online Repository at www.jaci-global.org. More derived data supporting the findings of this study are available from the corresponding authors on request.

## Results

### Annual publication and major research directions

There were 3907 studies on CRS and NPs between January 1, 2003, and December 31, 2022, including 3266 “articles” and 641 “reviews.” The number of publications on CRS and NPs steadily increased in the past 2 decades.

**Fig 1:**
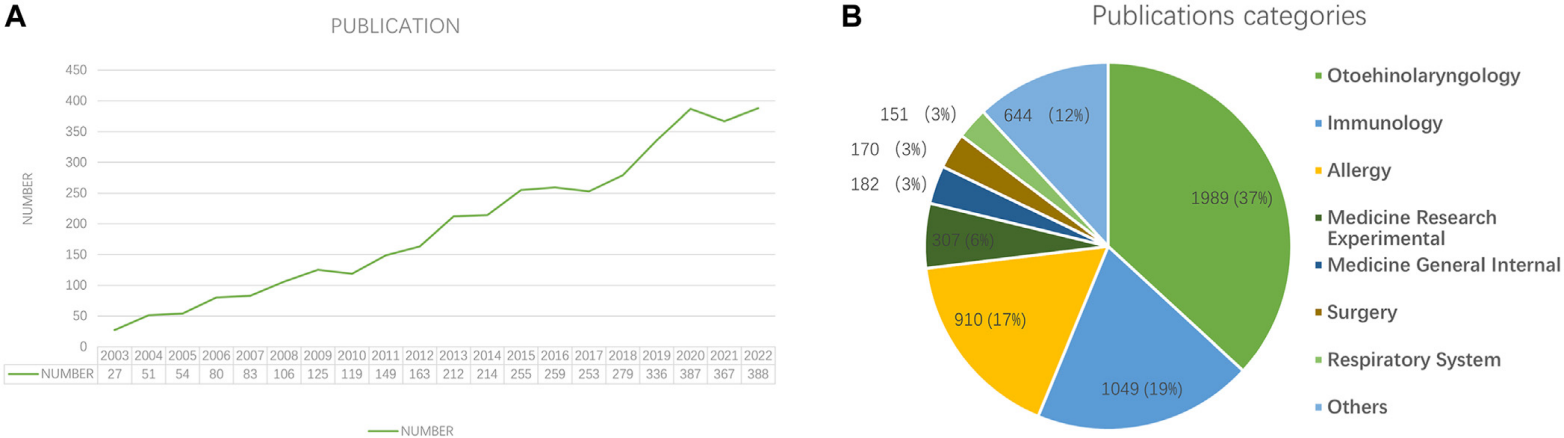
Publications output over 20 years. **A,** Annual publication number of research on CRS and NPs. **B,** The number and proportion of publications in different research categories.

As shown in Fig 1, *A*, this field attracted the attention of scientific researchers at an early stage. Of the studies, 27 were published in 2003. From 2003 to 2020, the number of publications increased rapidly. The publications have increased 13.3 times (27 in 2003 and 387 in 2020). Starting from 2020, the publication trend in the last 3 years (2020-2022) had been relatively stable.

We classified the 3907 studies on CRS and NPs (Fig 1, *B*) and found that “otorhinolaryngology,” “immunology,” and “allergy” were the 3 primary research directions in the research.

### Most influential country and institution

These publications came from 82 countries and 3139 institutions. We set the publication threshold to 10 publications, and 43 countries met the criteria. The top 10 countries were distributed in Asia, North America, and Europe, with most being European countries (n = 5). The country with the highest number of publications was the United States (n = 1248 [33.5%]), followed by China (n = 631 [17.0%]), Korea (n = 293 [7.9%]), and Belgium (n = 272 [7.3%]). During these 20 years, the total number of citations of publications published in the United States ranked first with 39,027 citations (Table I).

**Table I:** Top 10 countries in terms of the number of publications and citations

Rank	Country	Documents	Citations
1	United States (North America)	1,248	39,027
2	China (Asia)	631	11,065
3	South Korea (Asia)	293	5,276
4	Belgium (Europe)	272	17,501
5	Japan (Asia)	257	7,726
6	Italy (Europe)	247	6,129
7	England (Europe)	237	12,219
8	Germany (Europe)	217	8,170
9	Canada (North America)	173	6,273
10	Sweden (Europe)	145	8,381

**Fig 2:**
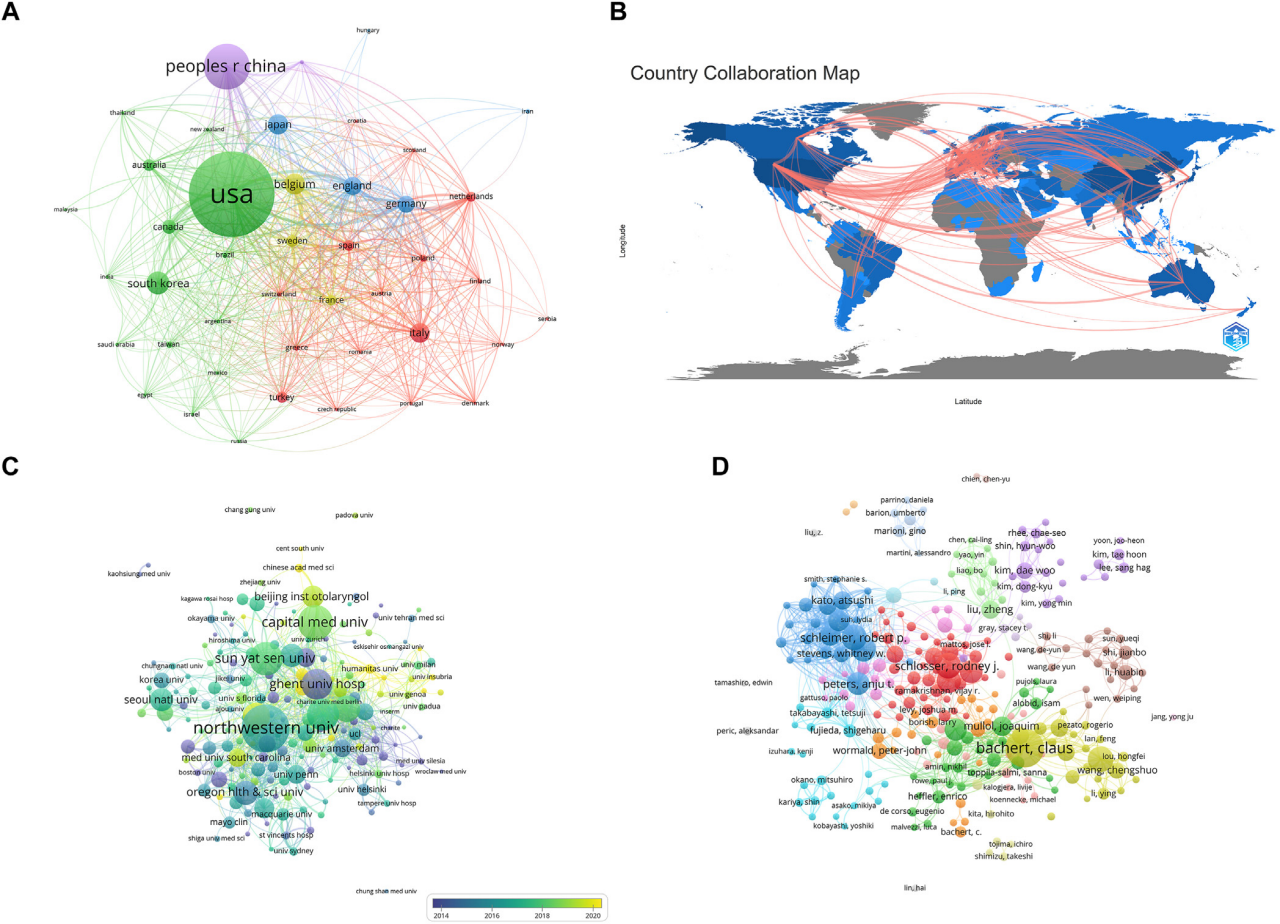
The collaborative networks between different units. **A,** Intercountry cooperation network. The color represents the cluster. **B,** Geographical distribution of intercountry cooperation. Each connecting line represents 5 collaborative publications between 2 countries. The color represents the number of publications. **C,** Interinstitutional cooperation network. The color represents the average year of publication. **D,** Author collaboration network.

**Supplementary Figure E1:**
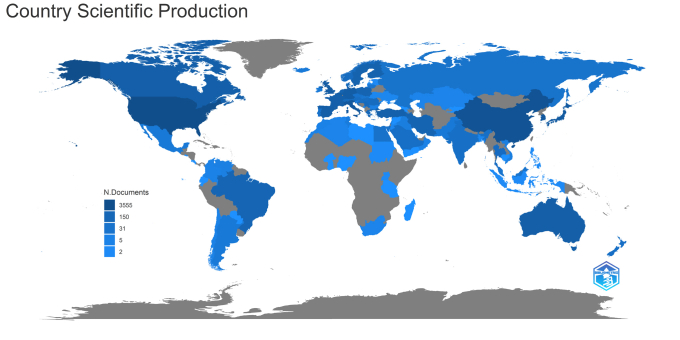


After selection based on the aforementioned publication thresholds, we visualized the number of publications and intercountry collaboration networks for these 43 countries (Fig 2, *A*). For example, the United States had close collaborative relationships with Canada, England, and Germany. In addition, we mapped the geographical distribution of intercountry collaborations (Fig 2, *B*) and the publication density (see Fig E1 in this article’s Online Repository at www.jaci-global.org).

As with these publication thresholds, 190 of the 3139 institutions met our criteria. The top 10 institutions were also located in Asia, North America, and Europe, mainly in the United States (n = 3) and China (n = 3). Northwestern University (n = 1248) was the top institution in terms of the number of publications, followed by Capital Medical University (n = 631) and Sun Yat-sen University (n = 293). Meanwhile, Northwestern University also achieved the highest citations. We analyzed the average year of the publications for the top 10 institutions, with larger values for Harvard Medical School (2019.4316), Capital Medical University (2018.4385), and Karolinska Institute (2018.043) (see Table E1 in this article’s Online Repository at www.jaci-global.org).

Similarly, we constructed a collaboration network among the 190 institutions and visualized the average year of the publications from different institutions (Fig 2, *C*). Most institutions with high publication volume collaborated with other institutions, especially within the same country. Some had many collaborating institutions, such as Sun Yat-sen University.

### Most impactful authors

A total of 12,894 authors had published in the research of CRS and NPs in the past 2 decades. Among them, 265 authors had published 10 or more publications. Calculated by the Price law, *m*_p_ = 9.41, and 265 core authors with 10 or more publications were counted. Table E2 (in the Online Repository available at www.jaci-global.org) provides the top 5 authors regarding the number of publications. The author with the most publications was C. Bachert, who had published 158 articles cited 10,093 times. Furthermore, we noted that L. Zhang had a significantly later average publication year than the other 4 professors and remained active in research related to CRS and NPs in recent years.

We drew a visualized collaboration network of these 265 authors (see Fig 2, *D*). The same color represents a cluster of authors with collaborative relationships, such as the collaboration among C. Bachert, W. Chengshuo, L. Hongfei, and others.

**Supplementary Figure E2:**
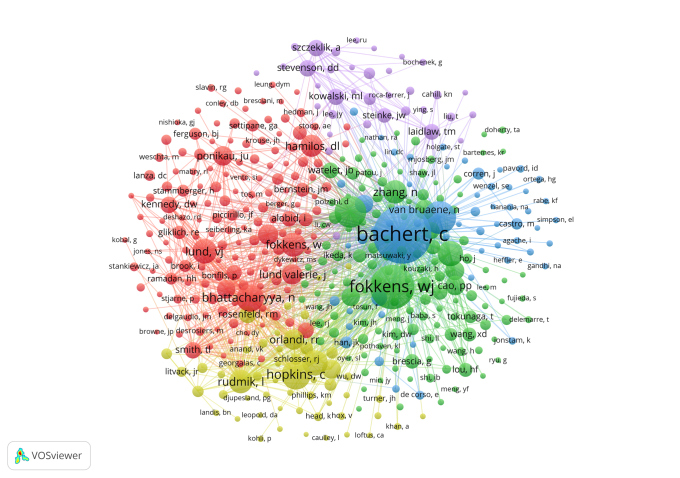


Authors with 50 or more co-citations were screened. The most co-cited author was C. Bachert (n = 2808), followed by W. J. Fokkens (n = 1767) and P. Gevaert (n = 1061) (see Table E3 in this article’s Online Repository at www.jaci-global.org). Close collaboration among the co-cited authors was prevalent (see Fig E2 in this article’s Online Repository at www.jaci-global.org).

### Most impactful journals

A total of 428 journals published publications related to CRS and NPs. We filtered them by the number of publications (≥5) and citations (≥50), and a total of 83 journals were eligible.

The journal with the highest number of publications was the *International Forum of Allergy & Rhinology* (n = 365; impact factor [IF] = 5.43, Q1), with an average publication year of 2016.7671, followed by the *American Journal of Rhinology & Allergy* (n = 318; IF = 2.30, Q2), the *Journal of Allergy and Clinical Immunology* (n = 188; IF = 14.29, Q1), and *Rhinology* (n = 188; IF = 6.63, Q1) (see Table E4 in this article’s Online Repository at www.jaci-global.org). Among the top 10 journals with the most publications, the *Journal of Allergy and Clinical Immunology: In Practice* (n = 2019.6389; IF = 11.02, Q1), the *International Forum of Allergy & Rhinology* (n = 2016.7671; IF = 5.43, Q1), and the *European Archives of Oto-rhino-laryngology* (n = 2016.0061; IF = 3.24, Q2) had an average later year of publication. These journals have published more relevant articles in recent years. In addition, according to our screening criteria, the most cited journal was the *Journal of Allergy and Clinical Immunology* (n = 13,739; IF = 14.29, Q1), with an average of 73 citations per article, followed by the *International Forum of Allergy & Rhinology* (n = 7,813; IF = 5.43, Q1) and *Allergy* (n = 6,880; IF = 14.71, Q1) (Table II).

**Table II:** Ranking of journals (in descending order by citations)

Rank	Journal	IF (Q)	Documents	Citations	Average publication year
1	*Journal of Allergy and Clinical Immunology*	14.29 (Q1)	188	13,739	2015.9628
2	*International Forum of Allergy & Rhinology*	5.43 (Q1)	365	7,813	2016.7671
3	*Allergy*	14.71 (Q1)	118	6,880	2014.5254
4	*Laryngoscope*	2.97 (Q2)	180	6,621	2013.2167
5	*American Journal of Rhinology & Allergy*	2.30 (Q3)	318	6,177	2015.1761
6	*Rhinology*	6.63 (Q1)	188	4,994	2015.0638
7	*Otolaryngology-Head and Neck Surgery*	5.59 (Q1)	102	3,333	2012.8725
8	*American Journal of Rhinology*	—	58	2,481	2006.6207
9	*Current Allergy and Asthma Reports*	4.92 (Q2)	83	1,986	2014.012
10	*Clinical and Experimental Allergy*	5.40 (Q2)	54	1,915	2014.7963

**Supplementary Figure E3:**
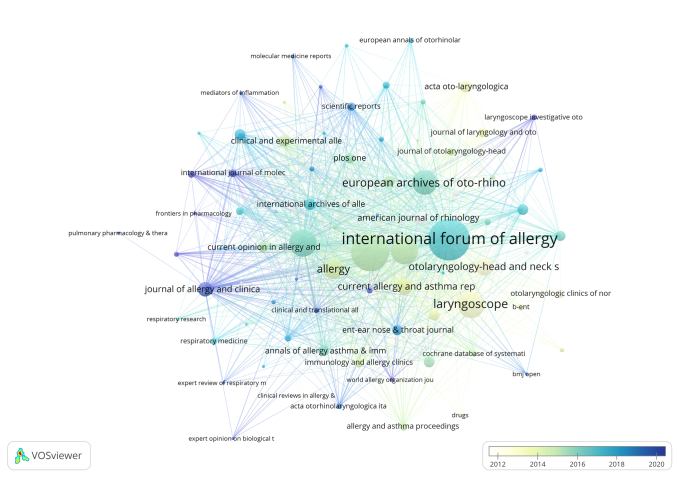


We plotted the interjournal citation network of these 83 journals and distinguished their average publication year by color, showing the citation relationship between different journals (see Fig E3 in this article’s Online Repository at www.jaci-global.org). For example, the *International Forum of Allergy & Rhinology* and the *European Archives of Oto-rhino-laryngology* had a strong citation relationship.

**Fig 3:**
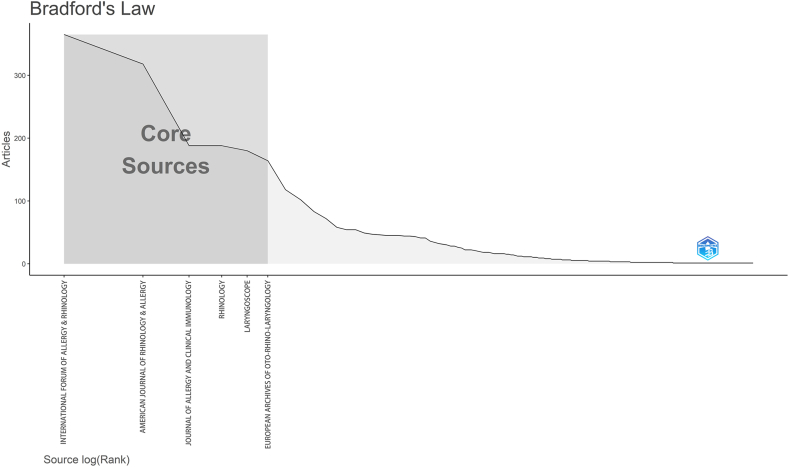
The core sources journals (according to the Bradford law) and the citation relationships between journals.

According to the Bradford law,^19^^,^^20^ evidence of the regularity of the distribution of scientific journals, it is always a small number of journals that contribute to the core sources in a field. We used the Bibliometrix R package to calculate the leading contributing journals in the research of CRS and NPs. These were the *International Forum of Allergy & Rhinology* (IF = 5.43, Q1), the *American Journal of Rhinology & Allergy* (IF = 2.30, Q2), the *Journal of Allergy and Clinical Immunology* (IF = 14.29, Q1), *Rhinology* (IF = 6.63, Q1), *Laryngoscope* (IF = 2.97, Q2), and the *European Archives of Oto-rhino-laryngology* (IF = 3.24, Q2) (Fig 3).

### Most widely cited references

From 2003 to 2022, there were 58,708 co-cited references in studies related to CRS and NPs, and we screened 241 references using a co-citation count of 50 or higher as the screening criterion.

We listed the top 10 most co-cited references (see Table E5 in this article’s Online Repository at www.jaci-global.org). The highest number of co-citations was published in *Rhinology* by Fokkens et al (European Position Paper on Rhinosinusitis and Nasal Polyps 2012 [EPOS 2012]^21^) with 1323 co-citations. In 2020, this article was updated and is now the European Position Paper on Rhinosinusitis and Nasal Polyps 2020 (EPOS 2020),^1^ the most authoritative and complete guide on CRS, followed by articles published by Lund et al^22^ (n = 459) and Van Zele et al^23^ (n = 428), both of which were published earlier, in 1993 and 2006, respectively. They illustrated the staging in rhinosinusitis and the differences in the cellular and media profiles of different types of CRS, respectively. These are both seminal studies in the research of CRS and NPs, focusing on the endophenotypic and immunologic features of CRS. The article with the fourth highest number of co-citations was published in 2016, the most recent of the top 10 co-cited references. Researchers analyzed IL-5, IFN-γ, IL-17A, TNF-α, IL-22, IL-1β, IL-6, IL-8, eosinophilic cationic protein, myeloperoxidase, TGF-β1, IgE, specific IgE, and albumin in the patient tissue samples and classified the inflammatory endotype of CRS by immunomarkers.^23^ This article generated discussion throughout the CRS and NPs fields about further refining the CRS immunophenotypic delineation.

**Supplementary Figure E4:**
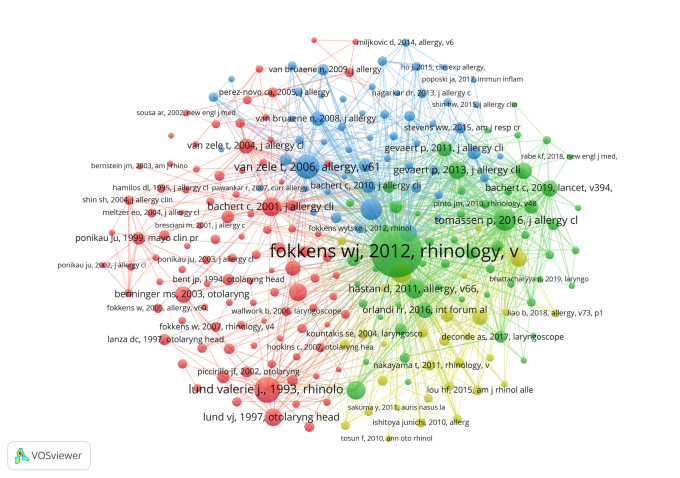


The top 10 co-cited references had a profound impact on the fundamental research. Interestingly, these 10 publications originated from only 4 journals, with the *Journal of Allergy and Clinical Immunology* (IF = 14.29, Q1) accounting for 5. At the same time, *Rhinology* (IF = 6.634, Q1) published the 2 references with the highest number of co-citations. We constructed a co-cited reference relationship network with co-cited citations 50 times or more (see Fig E4 in this article’s Online Repository at www.jaci-global.org).

### Keywords, hotspots, and trend topics

Author keywords and keywords plus for 3907 publications were included in our analysis.

By analyzing the author keywords of the publications of CRS and NPs during the past 20 years, we obtained 5138 author keywords, of which 233 appeared 10 times or more. Then we sorted these author keywords according to the average publication year of the corresponding articles and counted the occurrences and the average citations (see Table E6 in this article’s Online Repository at www.jaci-global.org). On the basis of these, we made a reasonable judgment of the frontiers and hotspots in the research of CRS and NPs.

As shown in Table E6, we presented the 20 author keywords with the latest average publication year, which represented the frontiers of research in this field in the last 3 years, with “Covid-19,” “biologics,” “benralizumab,” “dupilumab,” and “reslizumab” in the top 5. Covid-19 attracted significant attention throughout the medical field as a currently prevalent infectious disease. In addition, the other 4 author keywords in the top 5 indicated that the research of CRS and NPs focused on biologic treatments. Benralizumab, dupilumab, and reslizumab are mAbs to IL-5R, IL-4R, and IL-5, respectively, the biologics currently approved worldwide for allergic diseases such as asthma and atopic dermatitis.^24^^,^^25^ Among them, dupilumab was approved for CRS treatment in 2019. It proved that biologics and mAbs were attracting much attention from researchers in CRS and NPs.^26^^,^^27^ In addition, we highlight the author keywords with more than 50 occurrences. We found that “biologics” (n = 84), “CRSwNP” (n = 82), “dupilumab” (n = 77), “type 2 inflammation” (n = 69), and “omalizumab” (n = 55) showed a high number of occurrences in the frontier articles, which may mean that these are the hotspots of research in recent years.

**Fig 4:**
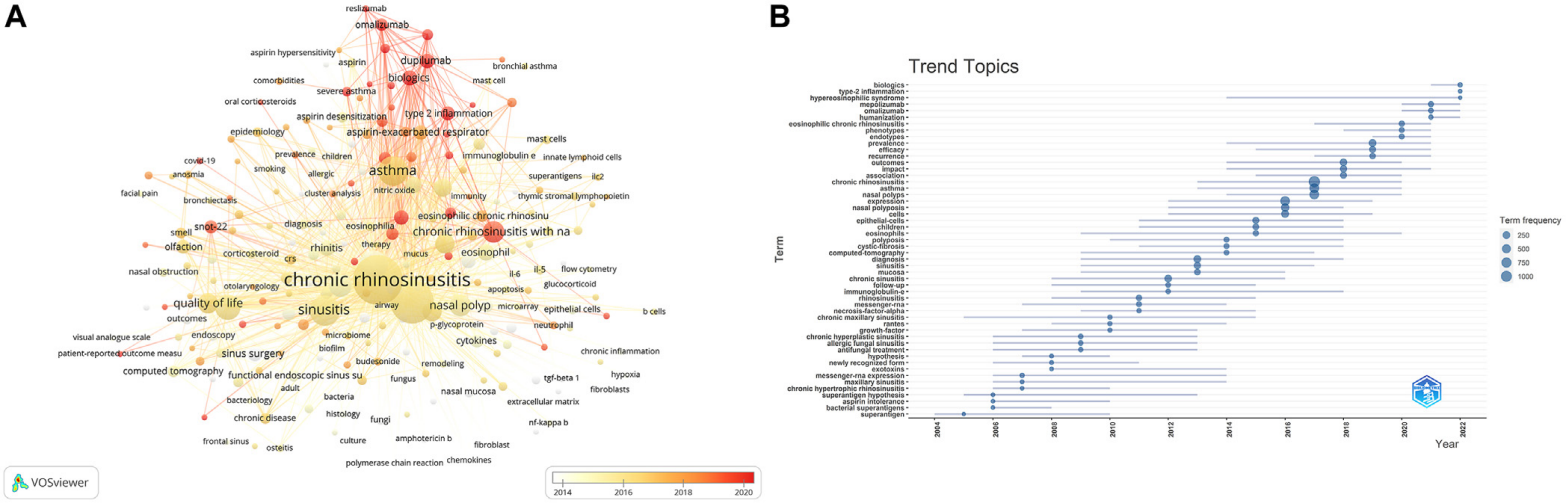
Inter-keywords network and trend topics. **A,** The relationship network between authors’ keywords. **B,** Trending topics analysis based on keywords plus.

**Supplementary Figure E5:**
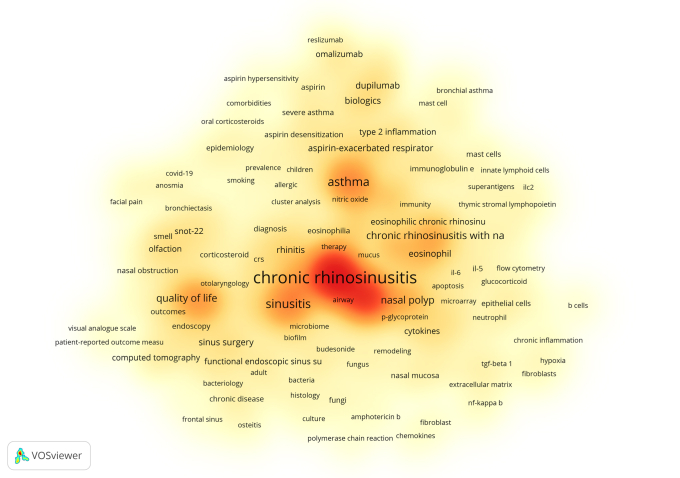


We plotted the hotspot diagram of author keywords with 10 or more occurrences (see Fig E5 in this article’s Online Repository at www.jaci-global.org) and the relationship network diagram between these keywords (Fig 4, *A*). The hotspot diagram can visually determine the frequency of keyword occurrences, with darker colors representing higher occurrences. In the keywords network diagram, a thicker line means a stronger connection between the 2, and the color represents the average occurrence year of that keyword.

**Supplementary Figure E6:**
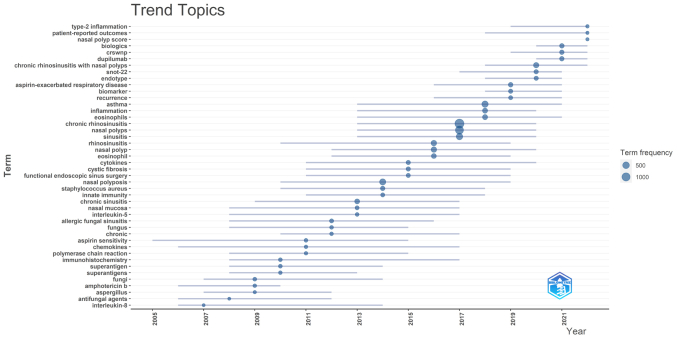


In addition, we organized trend topics on the basis of authors’ keywords (see Fig E6 in this article’s Online Repository at www.jaci-global.org) to visualize how research trends had changed over the 20 years, with the size of the circles representing the frequency of the corresponding topics. As we can see, “type 2 inflammation,” “patient-reported outcomes,” “nasal polyp score,” and “biologics” were the latest trend topics.

In addition, we built a trending topics analysis based on keywords plus (Fig 4, *B*) to provide a more comprehensive keyword analysis. Keywords plus can exclude the subjective factors of authors and reflect the main content discussed in publications through machine analysis. Fig 4, *B*, constructed on the basis of keywords plus, provides a more objective reflection of trending topics than Fig E6, which was constructed on the basis of authors’ keywords. The results showed that the most recent trending topics were “biologics,” “type 2 inflammation,” “hypereosinophilic,” “mepolizumab,” and others (more information can be found in the table). As we expected, the results of the keyword analysis were mutually corroborative. Researchers have recently focused on topics related to type 2 inflammation, biologics, and disease outcomes. It further strengthened the credibility of the data.

**Supplementary Figure E7:**
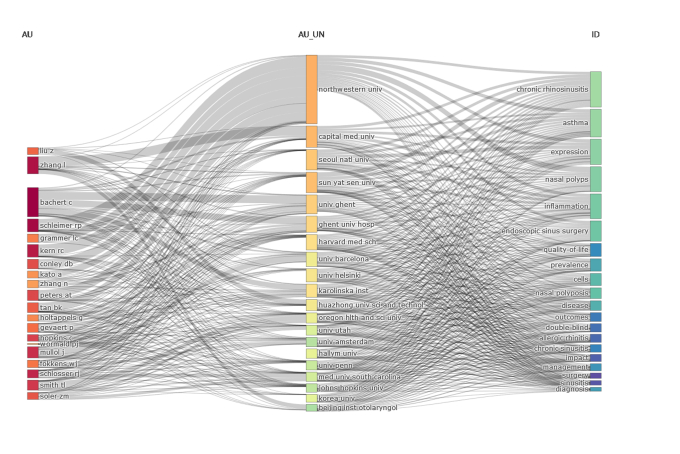


We combined affiliations, authors, and keywords plus to draw a Sankey diagram (see Fig E7 in this article’s Online Repository at www.jaci-global.org). The Sankey diagram can show the relationship between these 3 topics. The thickness of the lines represents the connection between the 2; for example, C. Bachert mainly worked with Ghent University, Ghent University Hospital, and Karolinska Institute. Northwestern University had more studies on NPs.

## Discussion

### General information

In the present study, we reviewed the current research in the research of CRS and NPs by bibliometrics for the first time and analyzed the publications in the last 20 years (2003-2022) to obtain a comprehensive overview of this research. We observed that (1) the United States and Northwestern University were the most influential country and institution with the most articles and the highest citations; (2) C. Bachert was the scholar with the most articles and the highest co-cited citations; (3) the *International Forum of Allergy & Rhinology* had the highest number of articles and the *Journal of Allergy and Clinical Immunology* had the highest number of citations; (4) the endotype and immune characteristics of CRS and NPs had received long-term and extensive attention; and (5) “Covid-19,” “biologics,” and “type 2 inflammation” were currently the top research hotspots. These findings are helpful for researchers to understand the current state and grasp potential directions and trending research topics on CRS and NPs.

### Results interpretation and research directions mining

#### Development situation

This research chose to analyze the Web of Science database because it is one of the most authoritative databases. CRS and NPs had received attention from researchers at an early stage, which may be related to the prevalence of this disease. From 2003 to 2020, global research on CRS and NPs grew substantially, with a generally stable growth rate. The average annual increase in publications for these 18 years was 20, with an annual average of 175.1 publications. Moreover, from 2020 to 2022, the growth reached a plateau, with an annual average of 380.7 publications. The peak and plateau of publication over the past 3 years indicated that the study was receiving sustained attention from researchers and was in a steady state of development and research.

The main subject area related to CRS and NPs was not only otolaryngology but also immunology and allergology. The immunologic mechanisms of CRS and NPs had received much attention from scholars, which may bring reference to some researchers.

#### Major contributing units

In CRS and NPs research, cooperation and linkages between countries were already intense. All countries with more than 10 publications were involved in international collaboration. Undoubtedly, it promoted the flourishing of CRS and NPs research. The United States and China led the way in the number of publications. Although European countries had fewer publications than these 2 countries, their contribution to the research cannot be ignored. Of the 10 institutions with the most publications, 3 were from Europe. And the Ghent University Hospital received the second most citations, following Northwestern University.

Of those institutions with more than 10 publications, we found that Chang Gung University, Padova University, and Chung Shan Medical University had never collaborated with other institutions, which may affect the continued development of these institutions. We call for broader collaboration among countries and institutions, leading to more CRS and NPs research breakthroughs.

A total of 428 journals published research on CRS and NPs over the 20 years, and the *International Forum of Allergy & Rhinology* (IF = 5.426, Q1) had the most publications, indicating that it is the most popular journal in this research. After compiling the top 10 journals regarding the number of publications, we noted that 6 journals are Q1 and 4 are Q2. The top 3 journals with the most citations were all high-impact journals in Q1. Although CRS and NPs belong to rhinological diseases, many articles were published in allergy and basic medicine journals in addition to rhinology journals.

#### Leading experts and related directions

The 5 authors with the most publications in this study were C. Bachert, R. P. Schleimer, R. J. Schlosser, L. Zhang, and R. C. Kern, with more than 80 publications. We found that C. Bachert also focused on biological studies in 2022, and the latest study in which he participated showed that omalizumab’s effectiveness is independent of *Staphylococcal* enterotoxin sensitization.^28^ Moreover, he also published as the first author of the study of the therapeutic effect of dupilumab and mepolizumab in patients with CRS.^27^^,^^29^ In addition, he constructed a decision tree model through the combination of nasal secretion biomarkers and clinical characteristics of patients with CRS to accurately define patients with type 2 CRS with NPs in a noninvasive way to give more rational treatment to patients in the clinic. ^30^ C. Bachert’s group has focused on various directions, including endotype clustering of patients with CRS on the basis of different molecules, surgery, biological therapies, pathophysiological mechanisms, and so on.^28^, ^29^, ^30^, ^31^, ^32^, ^33^, ^34^, ^35^ Recently, R. P. Schleimer has focused on frontier molecules and treatments such as retinoic acid, nasal secretion tissue plasminogen activator, and oral CRTH2 antagonist.^36^, ^37^, ^38^ However, R. J. Schlosser focused more on olfaction and the surgical treatment of CRS.^39^, ^40^, ^41^

C. Bachert was the author with the highest number of co-citations, followed by W. J. Fokkens and P. Gevaert. A high number of co-citations often means that the author has a large amount of seminal research in the field that has been cited several times by subsequent researchers. They were all involved in compiling the most co-cited publication in the research of CRS and NPs: European Position Paper on Rhinosinusitis and Nasal Polyps 2012. Interestingly, all 3 authors had recently published research on biologics as first authors^27^^,^^28^^,^^42^^,^^43^ (indeed, the updated EPOS 2020 is currently more relevant). As mentioned in the previous article, this fact reaffirmed the importance of biologics research in CRS and NPs research.

#### Highly cited articles and current hotspots

Studies with high co-citation counts often represent fundamental research in the field and are generally published earlier. Except for the EPOS, we summarized the 9 most co-cited articles, which can be divided into 3 categories: immune features and endotypes, clinical applications, and epidemiological analyses. Of these 9 articles, 6 explored the inflammatory patterns in CRS in different countries or different endotypes, and they collectively focused on the cell expression profile and immune marker profile of patients with CRS, such as T_H_1/T_H_2-skewed inflammation.^22^^,^^23^^,^^44^, ^45^, ^46^, ^47^ In EPOS 2020, experts defined CRS endotypes more precisely on the basis of primary or secondary, anatomical distribution, and dominant endotype.^1^ At the same time, experts suggest that type 2 inflammation–mediated CRS is more likely to accompany the development of NPs.^1^ Our results also reflected that the role of type 2 inflammation in the study of CRS and NPs had received much attention. In addition, 2 publications focused on clinical applications, respectively evaluating the effectiveness of the 22-item Sino-Nasal Outcome Test (SNOT; a derivative of the 20-item SNOT), a quality-of-life questionnaire for patients with CRS,^48^ and the clinical efficacy of omalizumab.^49^ Both the 20-item SNOT and the 22-item SNOT are widely used in clinical and scientific research. Moreover, the application of biologics in CRS has been a hot topic in recent years, and among the biologics, dupilumab was approved for treating CRS in 2019. Moreover, the last publication was the European international multicentre prevalence study of CRS.^50^ This article confirmed that the prevalence of CRS in Europe has significant geographic variation and is associated with smoking.^50^ It should be noted, however, that the value of an article is not reliably measured by co-citation alone, because the newly published articles will rank lower when analyzed in terms of citations independently. In contrast, many incoming articles have significantly affected frontier research.

By analyzing the timing and frequency of keyword appearances, we could obtain the current frontier research about CRS and NPs. Subsequently, we grouped the current frontier research hotspots into 3 sections: biologics, Covid-19, and inflammatory response and endotypes. As mentioned earlier, the inflammatory response and endotypes have been topics of long-standing interest since the investigation of the immune mechanisms of CRS, whereas biological agents have been at the forefront of research in recent years. In addition, Covid-19, an epidemic infectious disease in recent years, has received attention concerning various diseases, including CRS.

### Study limitations

The limitations of our study included that the analysis was performed using the Web of Science database and the language English, meaning that some publications from other databases and non-English publications were not included in our data analysis. It would cause us to omit parts of the publication and may have an impact on the results. But this is unlikely to change the main trends described in this article.

In addition to this, a small portion of the data had overlapping information because of technical limitations. Furthermore, a small part of the information in this article was duplicated or separated because of technical limitations. For example, “Ghent University” and “Ghent University Hospital” were recognized as different institutions in the analysis. However, after some effort, the analysis software could not combine these items, and human adjustments would have affected the accuracy of other data, such as the average publication year. Therefore, the original results have been retained in this article. Fortunately, such situations were rare and had a limited impact on our conclusions.

### Conclusions

This study provided an overview of studies on CRS and NPs from a bibliometric perspective. The number of relevant publications grew dramatically over 20 years, representing many scholars’ increasing interest in the area. The United States was the primary leader in this research. Northwestern University and C. Bachert were the most prolific institution and author, respectively. The *International Forum of Allergy & Rhinology* published the most number of articles, and the *Journal of Allergy and Clinical Immunology* was the most cited. Researchers can stay updated with the latest trends in this research by following them. Our study concluded that “Covid-19,” “biologics,” and “type 2 inflammation” were current research hotspots. These directions deserve attention from scholars. We expect that our study will facilitate closer communication and collaboration among scholars from different countries to jointly promote this scientific area’s progress. We expect our study to give scholars who are interested in the study of CRS and NPs, especially researchers initially exploring the disease, a quick overview of developments in this field from a macroscopic perspective and help researchers target the literature and research directions.

## Disclosure statement

This study was partly funded by the 10.13039/501100001809National Science Foundation of China (grant nos. 82171159 and 82073434).

Disclosure of potential conflict of interest: The authors declare that they have no relevant conflicts of interest.

## References

[1] Fokkens W.J., Lund V.J., Hopkins C., Hellings P.W., Kern R., Reitsma S. (2020). European Position Paper on Rhinosinusitis and Nasal Polyps 2020. Rhinology.

[2] Bleier B.S. (2020). Topical glucocorticoid treatment for chronic rhinosinusitis in the biologic era. Int Forum Allergy Rhinol.

[3] Simmonds J.C., Paz-Lansberg M., Scangas G., Metson R. (2022). Endoscopic sinus surgery for chronic rhinosinusitis: 22-item Sino-Nasal Outcome Test 5-year results. Int Forum Allergy Rhinol.

[4] Miglani A., Divekar R.D., Azar A., Rank M.A., Lal D. (2018). Revision endoscopic sinus surgery rates by chronic rhinosinusitis subtype. Int Forum Allergy Rhinol.

[5] Dietz de Loos D.A., Hopkins C., Fokkens W.J. (2013). Symptoms in chronic rhinosinusitis with and without nasal polyps. Laryngoscope.

[6] Akmal M., Hasnain N., Rehan A., Iqbal U., Hashmi S., Fatima K. (2020). Glioblastome multiforme: a bibliometric analysis. World Neurosurg.

[7] Devos P., Menard J. (2019). Bibliometric analysis of research relating to hypertension reported over the period 1997-2016. J Hypertens.

[8] Gienapp A.J., Pippenger W., McGregor A.L., Fulton S.P. (2022). Publications in pediatric epilepsy: using bibliometrics to determine readings in the field. J Child Neurol.

[9] Ahmad P., Slots J. (2000 2021). A bibliometric analysis of periodontology. Periodontol.

[10] Wilson M., Sampson M., Barrowman N., Doja A. (2021). Bibliometric analysis of neurology articles published in general medicine journals. JAMA Netw Open.

[11] Luo X., Zhong R., Wang X., Yang G., Jiang X., Peng Y. (2022). Twenty-year span of global acute pancreatitis trends: a bibliometric analysis. Pancreatology.

[12] van Eck N.J., Waltman L. (2010). Software survey: VOSviewer, a computer program for bibliometric mapping. Scientometrics.

[13] Pan X., Yan E., Cui M., Hua W. (2018). Examining the usage, citation, and diffusion patterns of bibliometric mapping software: a comparative study of three tools. J Informetrics.

[14] Synnestvedt M.B., Chen C., Holmes J.H. (2005). CiteSpace II: visualization and knowledge discovery in bibliographic databases. AMIA Annu Symp Proc.

[15] Wu F., Gao J., Kang J., Wang X., Niu Q., Liu J. (2022). Knowledge mapping of exosomes in autoimmune diseases: a bibliometric analysis (2002-2021). Front Immunol.

[16] Sun H.L., Bai W., Li X.H., Huang H., Cui X.L., Cheung T. (2022). Schizophrenia and inflammation research: a bibliometric analysis. Front Immunol.

[17] Ma D., Yang B., Guan B., Song L., Liu Q., Fan Y. (2021). A bibliometric analysis of pyroptosis from 2001 to 2021. Front Immunol.

[18] Zhou F., Zhang T., Jin Y., Ma Y., Xian Z., Zeng M. (2022). Developments and emerging trends in the global treatment of chronic rhinosinusitis from 2001 to 2020: a systematic bibliometric analysis. Front Surg.

[19] Venable G.T., Shepherd B.A., Loftis C.M., McClatchy S.G., Roberts M.L., Fillinger M.E. (2016). Bradford’s law: identification of the core journals for neurosurgery and its subspecialties. J Neurosurg.

[20] Brookes B.C. (1969). Bradford’s law and the bibliography of science. Nature.

[21] Fokkens W.J., Lund V.J., Mullol J., Bachert C., Alobid I., Baroody F. (2012). European Position Paper on Rhinosinusitis and Nasal Polyps 2012. Rhinol Suppl.

[22] Lund V.J. (1993). Mackay IS. Staging in rhinosinusitus. Rhinology.

[23] Van Zele T., Claeys S., Gevaert P., Van Maele G., Holtappels G., Van Cauwenberge P. (2006). Differentiation of chronic sinus diseases by measurement of inflammatory mediators. Allergy.

[24] Agache I., Beltran J., Akdis C., Akdis M., Canelo-Aybar C., Canonica G.W. (2020). Efficacy and safety of treatment with biologicals (benralizumab, dupilumab, mepolizumab, omalizumab and reslizumab) for severe eosinophilic asthma. A systematic review for the EAACI Guidelines—recommendations on the use of biologicals in severe asthma. Allergy.

[25] Seegräber M., Srour J., Walter A., Knop M., Wollenberg A. (2018). Dupilumab for treatment of atopic dermatitis. Expert Rev Clin Pharmacol.

[26] Siddiqui S., Bachert C., Chaker A.M., Han J.K., Hellings P.W., Peters A.T. (2022). AROMA: real-world global registry of dupilumab for chronic rhinosinusitis with nasal polyps. ERJ Open Res.

[27] Bachert C., Khan A.H., Hopkins C., Blaiss M.S., Soler Z.M., Nash S. (2022). Rapid and continuing improvements in nasal symptoms with dupilumab in patients with severe CRSwNP. J Asthma Allergy.

[28] Migueres N., Poirot A., Zhang N., Bachert C., de Blay F. (2023). Omalizumab effectiveness is independent of Staphylococcal enterotoxin sensitization. Respir Med Res.

[29] Bachert C., Sousa A.R., Han J.K., Schlosser R.J., Sowerby L.J., Hopkins C. (2022). Mepolizumab for chronic rhinosinusitis with nasal polyps: treatment efficacy by comorbidity and blood eosinophil count. J Allergy Clin Immunol.

[30] Wang Z., Wang Q., Duan S., Zhang Y., Zhao L., Zhang S. (2022). A diagnostic model for predicting type 2 nasal polyps using biomarkers in nasal secretion. Front Immunol.

[31] Förster-Ruhrmann U., Szczepek A.J., Pierchalla G., Fluhr J.W., Artuc M., Zuberbier T. (2022). Chemokine expression-based endotype clustering of chronic rhinosinusitis. J Pers Med.

[32] Plath M., Derycke L., Sand M., Van de Vyvere D., Delemarre T., Cavaliere C. (2023). Can patient-reported outcomes and inflammatory markers define endotype 2 in chronic rhinosinusitis without nasal polyps?. Ann Allergy Asthma Immunol.

[33] Wang X., Sima Y., Zhao Y., Zhang N., Zheng M., Du K. (2023). Endotypes of chronic rhinosinusitis based on inflammatory and remodeling factors. J Allergy Clin Immunol.

[34] Shen Y., Zhang N., Yang Y., Hong S., Bachert C. (2022). Local immunoglobulin E in nasal polyps: role and modulation. Front Immunol.

[35] Gomes S.C., Cavaliere C., Masieri S., Van Zele T., Gevaert P., Holtappels G. (2022). Reboot surgery for chronic rhinosinusitis with nasal polyposis: recurrence and smell kinetics. Eur Arch Otorhinolaryngol.

[36] Sakashita M., Takabayashi T., Imoto Y., Homma T., Yoshida K., Ogi K. (2022). Retinoic acid promotes fibrinolysis and may regulate polyp formation. J Allergy Clin Immunol.

[37] Chen C.L., Zhao J.F., Guo C.L., Pan L., Ma J., Wang Y.T. (2022). Nasal secretion tissue plasminogen activator: a novel effective predictor of nasal polyp recurrence. J Allergy Clin Immunol Pract.

[38] Price C.P.E., Guo A., Stevens W.W., Cousens L., Vu T.T., Suh L.A. (2022). Efficacy of an oral CRTH2 antagonist (AZD1981) in the treatment of chronic rhinosinusitis with nasal polyps in adults: a randomized controlled clinical trial. Clin Exp Allergy.

[39] Mattos JL, Hasan S, Schlosser RJ, Payne SC, Soler ZM. The association of gustatory dysfunction, olfactory dysfunction, and cognition in older adults [published online ahead of print December 23, 2022]. Int Forum Allergy Rhinol. https://doi.org/10.1002/alr.23126. 10.1002/alr.23126 PMC10412899 36562185

[40] Schlosser R.J., Dubno J.R., Eckert M.A., Benitez A.M., Gregoski M., Ramakrishnan V. (2022). Unsupervised clustering of olfactory phenotypes. Am J Rhinol Allergy.

[41] Pandrangi V.C., Mace J.C., Kim J.H., Geltzeiler M., Detwiller K.Y., Soler Z.M. (2023). Work productivity and activity impairment in patients with chronic rhinosinusitis undergoing endoscopic sinus surgery—a prospective, multi-institutional study. Int Forum Allergy Rhinol.

[42] Fokkens W., Trigg A., Lee S.E., Chan R.H., Diamant Z., Hopkins C. (2023). Mepolizumab improvements in health-related quality of life and disease symptoms in a patient population with very severe chronic rhinosinusitis with nasal polyps: psychometric and efficacy analyses from the SYNAPSE study. J Patient Rep Outcomes.

[43] Gevaert P., Lee S.E., Settipane R.A., Wagenmann M., Msihid J., Siddiqui S. (2023). Dupilumab provides early and durable improvement of symptoms in patients with chronic rhinosinusitis with nasal polyps. Clin Transl Immunol.

[44] Tomassen P., Vandeplas G., Van Zele T., Cardell L.O., Arebro J., Olze H. (2016). Inflammatory endotypes of chronic rhinosinusitis based on cluster analysis of biomarkers. J Allergy Clin Immunol.

[45] Cao P.P., Li H.B., Wang B.F., Wang S.B., You X.J., Cui Y.H. (2009). Distinct immunopathologic characteristics of various types of chronic rhinosinusitis in adult Chinese. J Allergy Clin Immunol.

[46] Zhang N., Van Zele T., Perez-Novo C., Van Bruaene N., Holtappels G., DeRuyck N. (2008). Different types of T-effector cells orchestrate mucosal inflammation in chronic sinus disease. J Allergy Clin Immunol.

[47] Bachert C., Gevaert P., Holtappels G., Johansson S.G., van Cauwenberge P. (2001). Total and specific IgE in nasal polyps is related to local eosinophilic inflammation. J Allergy Clin Immunol.

[48] Hopkins C., Gillett S., Slack R., Lund V.J., Browne J.P. (2009). Psychometric validity of the 22-item Sinonasal Outcome Test. Clin Otolaryngol.

[49] Gevaert P., Calus L., Van Zele T., Blomme K., De Ruyck N., Bauters W. (2013). Omalizumab is effective in allergic and nonallergic patients with nasal polyps and asthma. J Allergy Clin Immunol.

[50] Hastan D., Fokkens W.J., Bachert C., Newson R.B., Bislimovska J., Bockelbrink A. (2011). Chronic rhinosinusitis in Europe—an underestimated disease. A GA^2^LEN study. Allergy.

