# Supplementary material for: Visual and bibliometric analysis of chronic rhinosinusitis and nasal polyps
Source: J Allergy Clin Immunol Glob. 2024 Jan 18;3(2):100211. doi: 10.1016/j.jacig.2024.100211 (PMC10899047; doi:10.1016/j.jacig.2024.100211)
Supplement: Supplementary data [file mmc1.docx]

**Table E1**

|  | **Institution** | **Documents** | **Citations** | **Avg. pub. year** |
| --- | --- | --- | --- | --- |
| **1** | **Northwestern University (The United States)** | **191** | **8916** | **2016.2775** |
| **2** | **Capital Medical University (China)** | **130** | **3068** | **2018.4385** |
| **3** | **Sun Yat-sen University (China)** | **123** | **2314** | **2017.9268** |
| **4** | **Ghent University (Belgium)** | **116** | **7107** | **2017.5172** |
| **5** | **Ghent University Hospital (Belgium)** | **111** | **8459** | **2014.2072** |
| **6** | **Harvard Medical School (The United States)** | **95** | **1684** | **2019.4316** |
| **7** | **Karolinska Institute (Sweden)** | **93** | **5343** | **2018.043** |
| **8** | **Oregon Health and Science University**  **(The United States)** | **84** | **2852** | **2015.2976** |
| **9** | **Seoul National University (South Korea)** | **78** | **1633** | **2016.9872** |
| **10** | **Huazhong University of Science and Technology (China)** | **77** | **2440** | **2016.4805** |

**Table E2**

| **Author** | **Documents** | **Citations** | **Avg. pub. year** |
| --- | --- | --- | --- |
| **Bachert, Claus** | **158** | **10093** | **2016.6266** |
| **Schleimer, Robert P.** | **85** | **4629** | **2016.4824** |
| **Schlosser, Rodney J.** | **85** | **1922** | **2016.1765** |
| **Zhang, Luo** | **84** | **2522** | **2019.0238** |
| **Kern, Robert C.** | **80** | **3989** | **2016.0125** |

**Table E3**

| **Co-Cited Author** | **Citations** |
| --- | --- |
| **Bachert, C** | **2808** |
| **Fokkens, WJ** | **1767** |
| **Gevaert, P** | **1061** |
| **Van zele, T** | **1009** |
| **Hopkins, C** | **852** |
| **Bhattacharyya, N** | **760** |
| **Soler, ZM** | **672** |
| **Stevens, WW** | **667** |
| **Lund, VJ** | **665** |
| **Fokkens, W** | **627** |

**Table E4**

| **Rank** | **Journal** | **IF (Q)** | **Documents** | **Citations** | **Avg. pub. year** |
| --- | --- | --- | --- | --- | --- |
| **1** | **international forum of allergy & rhinology** | **5.43 (Q1)** | **365** | **7813** | **2016.7671** |
| **2** | **american journal of rhinology & allergy** | **2.30 (Q2)** | **318** | **6177** | **2015.1761** |
| **3** | **journal of allergy and clinical immunology** | **14.29 (Q1)** | **188** | **13739** | **2015.9628** |
| **4** | **rhinology** | **6.63 (Q1)** | **188** | **4994** | **2015.0638** |
| **5** | **laryngoscope** | **2.97 (Q2)** | **180** | **6621** | **2013.2167** |
| **6** | **european archives of oto-rhino-laryngology** | **3.24 (Q2)** | **164** | **1803** | **2016.0061** |
| **7** | **allergy** | **14.71 (Q1)** | **118** | **6880** | **2014.5254** |
| **8** | **otolaryngology-head and neck surgery** | **5.59 (Q1)** | **102** | **3333** | **2012.8725** |
| **9** | **current allergy and asthma reports** | **4.92 (Q2)** | **83** | **1986** | **2014.012** |
| **10** | **journal of allergy and clinical immunology-in practice** | **11.02 (Q1)** | **72** | **1577** | **2019.6389** |

**Table E5**

| **Rank** | **Co-cited reference** | **Citations** |
| --- | --- | --- |
| **1** | **Fokkens WJ, 2012, Rhinology, V50, P1, DOI 10.4193/rhin20.600** | **1323** |
| **2** | **Lund Valerie J., 1993, Rhinology (utrecht), V31, P183** | **459** |
| **3** | **Van Zele T, 2006, Allergy, V61, P1280, DOI 10.1111/j.1398-9995.2006.01225.x** | **428** |
| **4** | **Tomassen P, 2016, J Allergy Clin Immun, V137, P1449, DOI 10.1016/j.jaci.2015.12.1324** | **365** |
| **5** | **Cao PP, 2009, J Allergy Clin Immun, V124, P478, DOI 10.1016/j.jaci.2009.05.017** | **344** |
| **6** | **Zhang N, 2008, J Allergy Clin Immun, V122, P961, DOI 10.1016/j.jaci.2008.07.008** | **337** |
| **7** | **Hastan D, 2011, Allergy, V66, P1216, DOI 10.1111/j.1398-9995.2011.02646.x** | **305** |
| **8** | **Bachert C, 2001, J Allergy Clin Immun, V107, P607, DOI 10.1067/mai.2001.112374** | **286** |
| **9** | **Hopkins C, 2009, Clin Otolaryngol, V34, P447, DOI 10.1111/j.1749-4486.2009.01995.x** | **286** |
| **10** | **Gevaert P, 2013, J Allergy Clin Immun, V131, P110, DOI 10.1016/j.jaci.2012.07.047** | **261** |

**Table E6**

| **Keyword** | **Occurrences** | **Avg. pub. year** | **Avg. citations** |
| --- | --- | --- | --- |
| covid-19 | 12 | 2021.1667 | 12.6667 |
| biologic | 10 | 2021.1 | 12.6 |
| benralizumab | 28 | 2020.8929 | 11.5357 |
| **dupilumab** | **77** | **2020.8442** | **14.1039** |
| reslizumab | 13 | 2020.6154 | 17.8462 |
| **biologics** | **84** | **2020.6071** | **16.119** |
| type-2 inflammation | 10 | 2020.6 | 13.4 |
| **type 2 inflammation** | **69** | **2020.5652** | **24.4638** |
| monoclonal antibody | 15 | 2020.2667 | 7.8667 |
| patient-reported outcome measure | 10 | 2020 | 9.7 |
| severe asthma | 31 | 2020 | 15.129 |
| mepolizumab | 42 | 2019.9048 | 28.4286 |
| **crswnp** | **82** | **2019.8902** | **11.7073** |
| crssnp | 16 | 2019.875 | 18.9375 |
| endotypes | 39 | 2019.8462 | 40.9487 |
| chronic rhinosinusitis with nasal polyps (crswnp) | 19 | 2019.8421 | 13.7895 |
| monoclonal antibodies | 22 | 2019.7727 | 12.5455 |
| medical therapy of chronic rhinosinusitis | 11 | 2019.7273 | 11.8182 |
| **omalizumab** | **55** | **2019.7273** | **18.5636** |
| biologicals | 13 | 2019.6923 | 24.3846 |
